# Cleanliness

**Published:** 1891-02

**Authors:** 


### Cleanliness.

Personal purity of the dentist is a necessity. It is an egregious fault, and in fact, an almost criminal circumstance for a dentist to be filthy about his person or instruments. Cleanliness should be a banner word for the professional man whose contact with ladies and gentlemen is at all times demanded. In general the dentist should be decently dressed, not flashily, but rather, should wear good plain clothes. Clean linen bespeaks a clean operator. Odors emanating from the pores of the skin should not be covered with the quart of cologne. Baths through the week, and daily sponge baths throughout the year, removes offensive debris of the secretions of the body. In summer hot baths should be limited. Special attention should be paid to the feet. Their close confinement in leather make them warm and sweaty, and tiredness is easily produced. Shoes with heavy soles will lessen chances of the becoming tired, experience teaches this. A change of shoes is often beneficial, but better still is at noontime soak the feet in running water, or a bucketful of water to which has been added some ammonia and borax, or either alone. This will make one feel like new, and the afternoon's work will seem easier. Tight-fitting clothing should be avoided. A tight collar will interfere with bending the head, and at the same time pressure upon the muscles of the neck will retard the flow of blood, producing among other things disturbances of blood supply to the brain, and also have its effect upon the eyesight. Good feet are a part of a dentist's capital, and care should be taken that they are properly encased with good-fitting shoes that will in no way cause pain or weariness.

A good preparation and care of the body is conducive to a steady hand and good eyes.

Another factor that is of very great importance concerning the personal purity of the dentist is the hands. There is as much necessity to care for the hands as for instruments.

Filthy and criminal indeed is he who would operate in one patient's mouth, and then turn to another without proper cleansing.

Diseases may be carried with the hand as well as with an instrument, but the principal way of carrying infection is the finger nail. Of course it is not the nail that poisons, but something that is on, or under the nail. In seventy-eight examinations recently made in Vienna (says the *British Med. Journal*) of the subungual spaces, there were found of micrococci 36 kinds, of bacilli 18 kinds, of sarcinæ 3 kinds, and common mould spores were very often present.

Personal cleanliness s'ould be insisted on in everybody, but how necessary it is for the dentist.

Under this heading might properly come the offensiveness of a liquored breath, or tobacco stench. Whisky ruins a good dentist, and tobacco will make him nervous. Leave whisky, women, and tobacco alone is wholesome advice for all. These and other matters of minor detail are things that every dentist should observe and study. W.

